# Expression and Clinical Significance of Cytochrome 1B1 in Bone Sarcomas

**DOI:** 10.3390/ph18101559

**Published:** 2025-10-16

**Authors:** Belal Al-zu’bi, Fatemah OFO Alshammari, Randa AlQaisi, Jaber H. Jaradat, Marwan Herzallah, Mohannad Ja’Awin, Anas O. Satari, Yousef M. Al-saraireh, Mohammad Salem Hareedy

**Affiliations:** 1 Department of Special Surgery, Faculty of Medicine, Mutah University, P.O. Box 7, Al-Karak 61710, Jordan; jaberjaradat2002@gmail.com (J.H.J.); 120201503027@mutah.edu.jo (M.H.); 420201503769@mutah.edu.jo (M.J.); 2 Department of Medical Laboratory Technology, Faculty of Health Sciences, The Public Authority for Applied Education and Training, Shuwaikh, Kuwait City 15432, Kuwait; fo.alowayed@paaet.edu.kw; 3 Department of Pediatrics, Faculty of Medicine, Mutah University, P.O. Box 7, Al-Karak 61710, Jordan; randasq@mutah.edu.jo; 4 Department of General Pediatrics, Maternity and Children’s Hospital at Al Bashir Hospital, Amman 11151, Jordan; satari@mutah.edu.jo; 5 Department of Pharmacology, Faculty of Medicine, Mutah University, P.O. Box 7, Al-Karak 61710, Jordan; 6 Department of Pharmacology, Faculty of Medicine, Assiut University, Assiut 71515, Egypt

**Keywords:** cytochromes, bone sarcomas, immunohistochemistry, transcriptomics, prognosis, drug sensitivity

## Abstract

**Background/Objectives:** Cytochrome 1B1 (CYP1B1) is overexpressed in several cancers, contributing to carcinogenesis, cancer progression, and chemoresistance. Despite its known oncogenic role, its expression in bone sarcomas remains unknown. **Methods:** This study assessed CYP1B1 expression in osteosarcoma and chondrosarcoma using immunohistochemistry on tissue microarrays and analyzed corresponding transcriptomic profiles from public RNA-seq datasets. Associations with clinicopathological features, survival, drug sensitivity, and protein–protein interaction networks were also investigated. **Results:** CYP1B1 was overexpressed in 72.3% of bone sarcomas (78% of osteosarcomas and 82.1% of chondrosarcomas) and was significantly underexpressed in normal bone (12.5%, *p* < 0.001). Importantly, high CYP1B1 expression was found in younger patients (≤34 years; *p* = 0.013), but no other associations with tumor grade, size, or metastasis were observed. The mean survival rate of CYP1B1-positive patients was insignificantly shorter than that of negative patients (58.8 vs. 62.8 months; *p* = 0.170). Although not confirmed in the multivariate analysis, CYP1B1-positive patients had poorer survival in the univariate analysis, which may reflect tumor aggressiveness rather than prognostic value. Transcriptomic data showed significantly lower CYP1B1 mRNA in osteosarcoma versus normal bone, suggesting post-transcriptional or translational regulation. Drug sensitivity analysis revealed both positive and negative correlations between CYP1B1 expression and response to various compounds in the GDSC dataset, highlighting potential therapeutic implications. **Conclusions:** Despite low mRNA levels, CYP1B1 protein is consistently and selectively overexpressed in bone sarcomas, particularly in younger patients. While not prognostic, its expression profile warrants further investigation and evaluation as a therapeutic target or diagnostic biomarker, especially in refractory or advanced cases.

## 1. Introduction

Cytochrome 1B1 (CYP1B1) is an enzyme that belongs to the CYP1 family, along with CYP1A1 and CYP1A2, and plays an important role in the metabolism of xenobiotics and endobiotics, including steroid hormones. This enzyme has recently gained significant attention for its ability to bioactivate aromatic compounds into carcinogens and hydroxylate estrogens [1,2,3]. Unlike other CYP1 enzymes, it is an extrahepatic enzyme and is more frequently overexpressed in many cancers, including lung, colon, breast, bladder, cervical, and prostate cancers. Importantly, its overexpression induces mutagenesis, oxidative stress, and drug resistance [3,4,5,6]. Such involvement in tumorigenesis positions CYP1B1 as a promising target for the development of novel cancer therapies.

Bone sarcomas are rare but aggressive tumors, representing fewer than 0.2% of cancers in humans, with an annual incidence of approximately 1.0 per 100,000 individuals. Their age incidence is bimodal, peaking in the second decade and beyond the age of 60 years [7,8]. Osteosarcoma and chondrosarcoma are the most prevalent histological types, accounting for approximately 35% and 25% of all bone sarcomas, respectively. Given advances in surgical procedures and chemotherapy regimens, the overall 5-year survival rate for localized bone sarcomas is about 68.5%, whereas it falls to less than 30% in recurrent and metastatic cases. Moreover, tumor cellular heterogeneity and chemoresistance remain crucial challenges in the treatment of bone sarcomas, causing significant variations in therapy responses [7,8,9,10,11]. Therefore, novel therapeutic strategies are urgently needed to overcome such a challenge, particularly for metastatic and refractory patients.

While the expression of certain CYPs, such as CYP3A4/5, has been investigated in bone sarcomas [12], the expression of CYP1B1 in bone sarcomas remains unclear. Therefore, this study aimed to characterize the expression patterns of CYP1B1 protein using immunohistochemistry in bone sarcomas, with a special focus on osteosarcoma and chondrosarcoma, and evaluate its association with relevant clinicopathological characteristics and patient outcomes. We also evaluated mRNA transcriptomic profiles using available datasets, mapped protein–protein interactions using the STRING database, predicted CYP1B1 protein functional domains using AlphaFold structural modeling and identified drug sensitivity associations using available pharmacogenomic datasets.

## 2. Results

### 2.1. Baseline Demographics and Clinicopathological Characteristics

The baseline demographics and clinical features of the 94 patients are presented in Table 1. Of the participants, 50 (53.2%) patient tissues with osteosarcoma and 28 (29.8%) patient tissues with chondrosarcoma were included in the study, while the rest, 16, were cases of healthy bone tissues (17%). The participants’ estimated average age was 34.1 ± 16.9 years. Among the participants, 55.3% (52 cases) were older than 34 years, and 44.7% (42 cases) were younger than 34 years. Regarding sex, male participants accounted for 66% (*n* = 62) of the participants, while the rest were female (32, 34%). Furthermore, approximately 74.4% of patients (58 cases) had a tumor of more than 8 cm (T2), whereas 25.6% (20 cases) had a tumor of less than 8 cm (T1). Additionally, only two (2.6%) cases presented with lymph node metastasis, and three (3.8%) cases presented with distant metastasis. Regarding tumor grade, most patients had grade 3 tumors (55, 70.5%), while the rest had grade 1 tumors (23, 29.5%). Moreover, tumor stage II accounted for most patient samples (50, 64.1%), although tumor stages I and IV accounted for 29.5% (23 cases) and 6.4% (5 cases), respectively.

### 2.2. CYP1B1 Protein Expression

**Figure 1:**
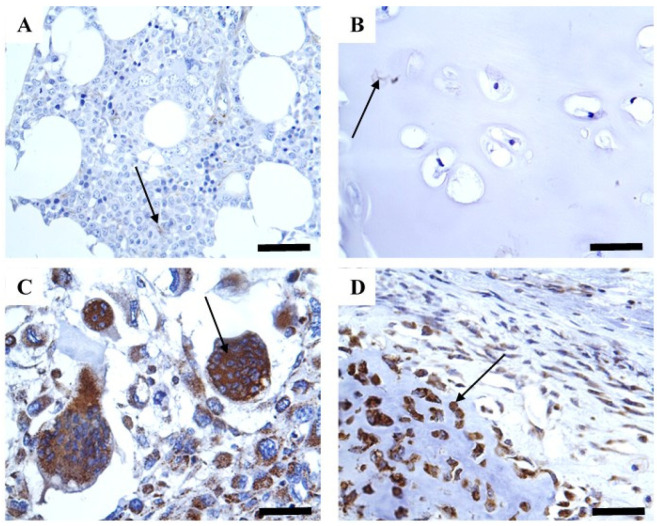
Expression of CYP1B1 in different types of bone sarcomas. (**A**) Minimal to no CYP1B1 expression was displayed in normal bone tissue; (**B**) Almost no CYP1B1 expression was detected in normal cartilage tissue; (**C**) Strong CYP1B1 expression was exhibited in osteosarcoma tissue; (**D**) High CYP1B1 expression was observed in chondrosarcoma tissue. Magnification (×400). Arrows indicate expression.

**Figure 2:**
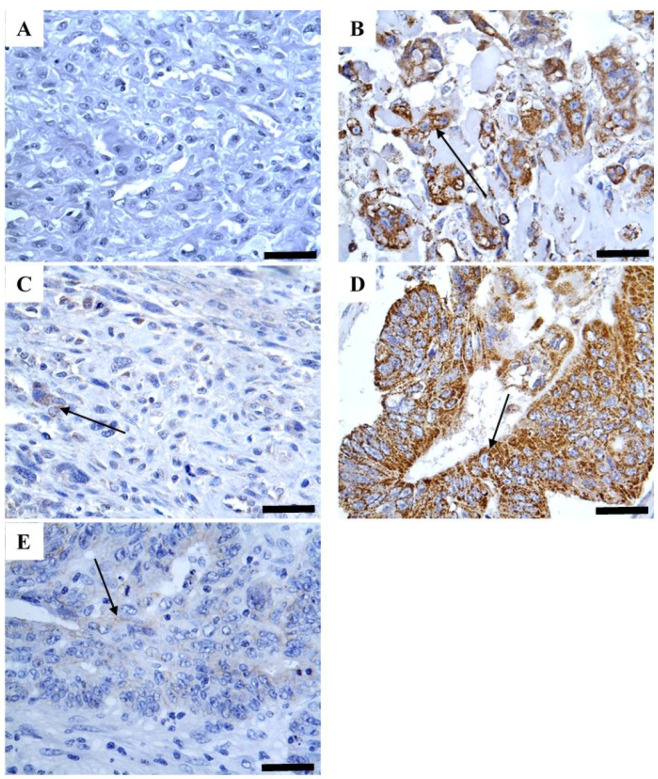
Expression of CYP1B1 in different kinds of experimental controls. (**A**) No CYP1B1 immunoreactivity was displayed in osteosarcoma tissue incubated with normal goat serum instead of CYP1B1 primary antibody (negative control); (**B**) CYP1B1 immunoreactivity was displayed in osteosarcoma tissue upon incubation with CYP1B1 primary antibody; (**C**) Almost no CYP1B1 immunoreactivity was seen in osteosarcoma tissue incubated with neutralized CYP1B1 primary antibody with corresponding protein peptide; (**D**) Strong CYP1B1 immunoreactivity was observed in colon tissue incubated with CYP1B1 primary antibody; and (**E**) Almost no CYP1B1 immunoreactivity was observed in colon tissue incubated with neutralized CYP1B1 primary antibody with corresponding protein peptide. Magnification (×400). Arrows indicate expression.

CYP1B1 was strongly detected in most bone sarcomas (79.5%), whereas normal samples displayed negligible expression (2 and 12.5%) (Table 1 and Figure 1). This expression was predominantly cytoplasmic immunoreactivity without obvious staining in the cell membrane and nucleus (Figure 1). Importantly, CYP1B1 expression was validated using colon cancer tissue as a positive control, and the inhibition of such expression was confirmed using a blocked CYP1B1 antibody (Figure 2). To investigate the associations between different patient characteristics and CYP1B1 expression, patient characteristics were categorized based on CYP1B1 expression status (CYP1B1-positive or CYP1B1-negative).

Data analysis showed a significant association between CYP1B1 expression and patient age and pathological tissue type (*p* < 0.05) (Table 1). A higher rate of CYP1B1 expression was found in patients younger than 34 years than in those older than this age. Moreover, there was a significant difference (*p* < 0.05) in CYP1B1 expression between the various bone sarcoma and normal bone tissue samples. CYP1B1 was highly expressed in osteosarcomas (39, 78%) and chondrosarcomas (23, 82.1%) compared to normal tissue samples (2, 2.5%). Despite the absence of a statistically significant difference in CYP1B1 expression, a trend of increasing CYP1B1 expression was observed with increasing tumor size, grade, and stage.

### 2.3. Analysis of CYP1B1 Gene Expression

**Figure 3:**
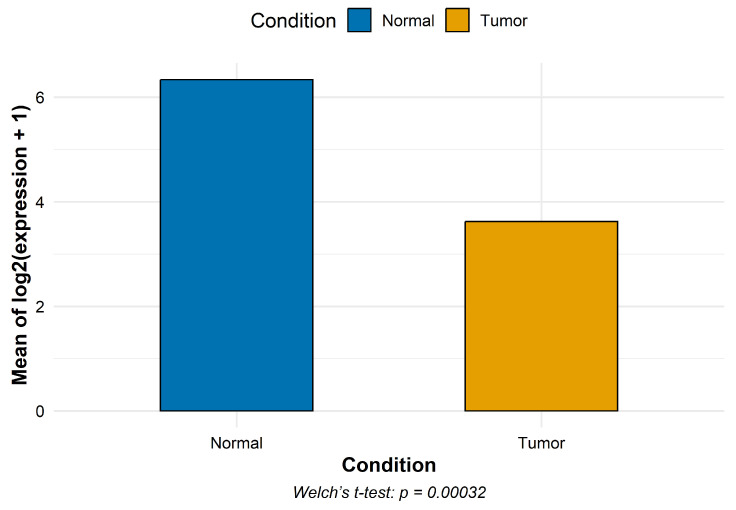
Mean log2(count + 1) expression of CYP1B1 in normal samples (*n* = 10) versus osteosarcoma (*n* = 12) samples.

CYP1B1 expression was markedly decreased in osteosarcoma compared to normal samples (mean log2(count + 1): 3.62 vs. 6.34), corresponding to an approximate six-fold decrease on the original count scale (Figure 3).

### 2.4. PPI Network and Clustering

The CYP1B1 interaction network comprised 11 nodes and 39 edges, significantly higher than the expected 10 edges, yielding a PPI enrichment *p*-value of 1.12 × 10^−11^. This indicated that the network had significantly more interactions than expected by chance. The average node degree was 7.09, and the average local clustering coefficient was 0.968, reflecting high connectivity and intermodular communication.

**Figure 4:**
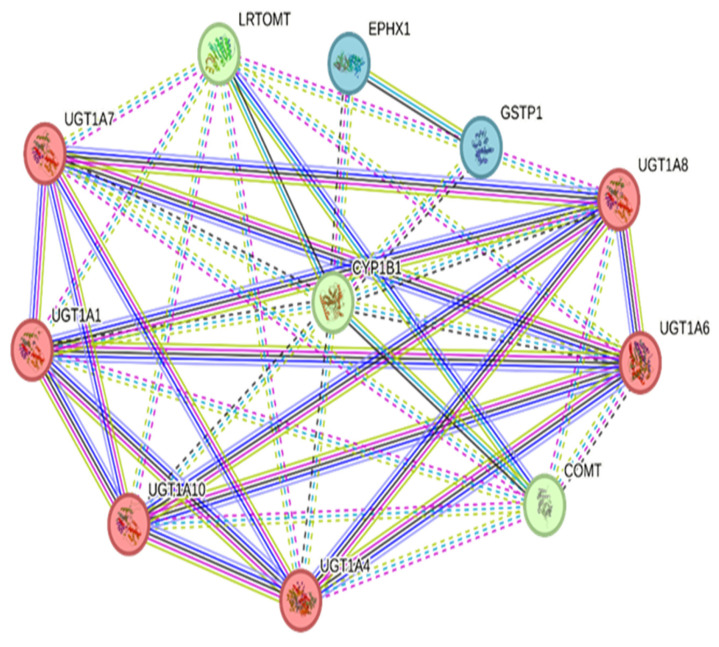
Protein–protein interaction (PPI) network of CYP1B1 and its functionally and physically associated proteins. Where each node represents a protein, and the edges denote known or predicted functional interactions. Nodes are color-coded based on k-means clustering analysis. Red cluster: UDP-glucuronosyltransferase family members (UGT1A1, UGT1A4, UGT1A6, UGT1A7, UGT1A8, UGT1A10), primarily involved in glucuronidation and phase II detoxification. Green cluster: CYP1B1, COMT, and LRTOMT, linked to catecholamine, estrogen, and xenobiotic metabolism. Blue cluster: EPHX1 and GSTP1, involved in epoxide hydrolysis and glutathione conjugation, respectively.

Three distinct protein clusters were identified using k-means clustering. Cluster 1 involved six genes, namely UGT1A1, UGT1A10, UGT1A4, UGT1A6, UGT1A7, and UGT1A8, which are members of the UDP-glucuronosyltransferase 1 family, known for their role in xenobiotic metabolism and glucuronidation of steroids and other toxic compounds. Cluster 2 included three genes, namely CYP1B1, COMT, and LRTOMT. COMT is a catechol-O-methyltransferase that detoxifies catecholamines and estrogens and acts downstream of CYP1B1-mediated hydroxylation (Figure 4). LRTOMT is a methyltransferase involved in neurotransmitter inactivation. Cluster 3 included two genes, EPHX1 and GSTP1, which are responsible for detoxification via epoxide hydrolysis and glutathione conjugation. These enzymes are implicated in the oxidative stress response and xenobiotic metabolism.

The high connectivity and modular structure of the CYP1B1 PPI network highlight its central role in detoxification, estrogen metabolism, and drug response pathways, which are critical in osteosarcoma and chondrosarcoma progression and drug resistance.

### 2.5. Analysis of Structural Prediction of Canonical CYP1B1 Using AlphaFold

**Figure 5:**
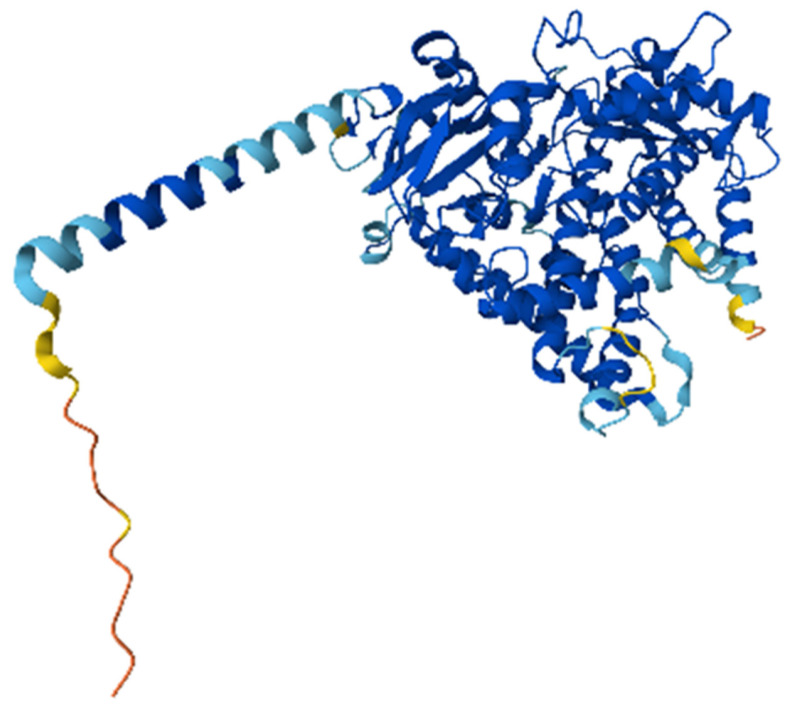
Predicted 3D molecular structure of the canonical CYP1B1 protein (UniProtKB Q16678) as generated by AlphaFold (AF-Q16678-F1). The color gradient represents residue-level confidence based on pLDDT scores: blue (very high confidence, >90), green (high, 70–90), yellow (low, 50–70), and orange/red (very low, <50). The heme-binding pocket and conserved domains are well resolved with high structural confidence.

**Figure 6:**
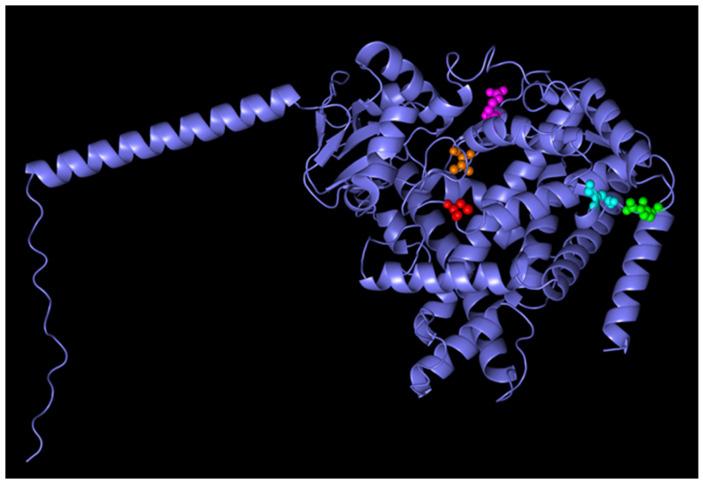
Structural mapping of CYP1B1 showing the heme-binding site and key residues. The heme-binding residue Cys470 is highlighted in red, the regiospecific site at residue 395 in orange, R368H in green, D374N in cyan, and R390H in magenta. The figure was generated using PyMOL (v3.1.6.1) based on UniProt data for CYP1B1 (Q16678).

The AlphaFold model of CYP1B1 revealed a well-structured protein composed of 543 residues, corresponding to the canonical isoform of the human CYP1B1 gene. The overall structural confidence was high, with most residues showing very high pLDDT scores (>90), indicating a reliable model suitable for downstream in silico analyses, such as docking or mutation effect prediction (Figure 5 and Figure 6). The predicted molecular structure highlights conserved features typical of cytochrome P450 enzymes, including the heme-binding domain and the monooxygenase fold. The predicted aligned error was low across the entire structure, suggesting minimal uncertainty in the inter-residue positioning. Two experimental PDB structures were previously deposited for CYP1B1 (UniProt Q16678); however, the AlphaFold model provides a complete full-length prediction, including regions that may be missing or unresolved in crystallographic datasets.

### 2.6. Analysis of Drug Sensitivity Correlation with CYP1B1 Expression

**Figure 7:**
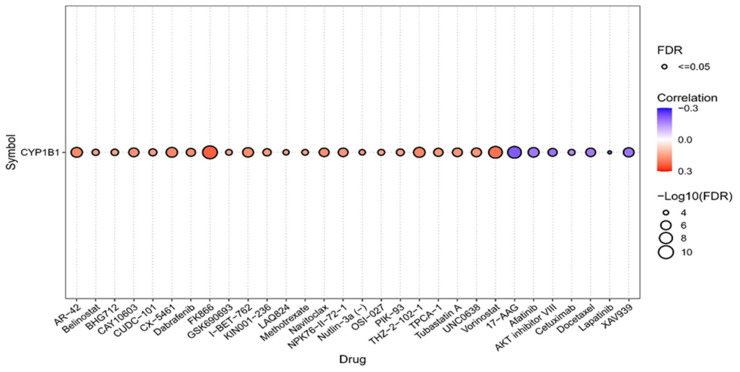
Correlation between GDSC drug sensitivity and CYP1B1 mRNA expression in pan-cancer using the GDSC database.

CYP1B1 expression was significantly correlated with the sensitivity of multiple compounds. Notably, AR-42, Belinostat, and CUDC-101 exhibited a positive correlation with CYP1B1, suggesting that increased expression is associated with greater sensitivity. In contrast, Afatinib, AKT inhibitor VIII, and Lapatinib showed a significant negative correlation, implying that high CYP1B1 expression may contribute to resistance. Overall, this analysis suggests that CYP1B1 expression may serve as a predictive biomarker for responsiveness or resistance to a subset of anticancer drugs, particularly HDAC inhibitors and EGFR/AKT-targeting agents (Figure 7).

### 2.7. Survival Analysis and Prognostic Value of CYP1B1 Expression

**Figure 8:**
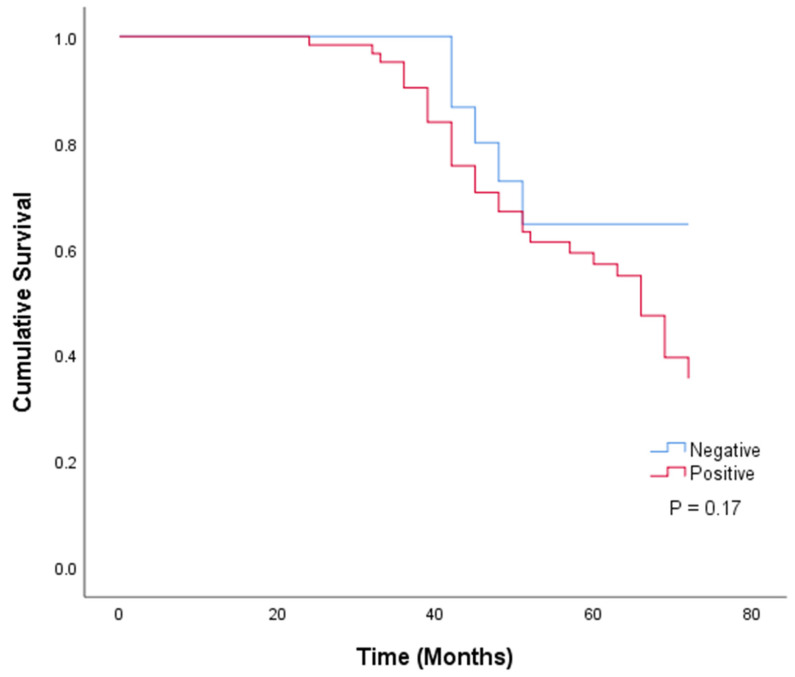
Kaplan–Meier curve illustrating cumulative survival for all the osteosarcoma and chondrosarcoma patients according to their CYP1B1 expression status. The *p*-value for the log-rank test is 0.170.

Only 78 bone sarcoma cases had survival data. Based on their CYP1B1 expression status, patients were divided into two groups: positive expression (79.5%, 62 patients) and negative expression (20.5%, 16 patients) groups. The Kaplan–Meier curve and log-rank test were used to analyze patient survival data regarding patient groups. Data analysis revealed that patients with positive CYP1B1 expression had a lower survival rate than those with negative CYP1B1 expression. However, no statistically significant difference was observed between the two groups (*p* = 0.17) (Figure 8). However, when the prognostic value of CYP1B1 was analyzed using univariate Cox regression, the overall survival rate was significantly affected by CYP1B1 expression (*p* = 0.039; HR = 1.335, 95% CI = 1.014–1.758), along with other parameters such as age, lymph node metastasis, distant metastasis, grade, and stage, implying poor survival (Table 2). Interestingly, the prognostic value of CYP1B1 in bone sarcomas was not independent when analyzed using multivariate Cox regression analysis. However, other parameters, such as age, tumor size, and grade, were independent prognostic predictors for bone sarcomas.

## 3. Discussion

To investigate the biochemical and clinical value of CYP1B1 in bone sarcomas, we analyzed its expression in osteosarcoma and chondrosarcoma tissues relative to normal bone tissues. CYP1B1 was found to be significantly overexpressed in bone sarcomas (osteosarcoma, 78%; chondrosarcoma, 82.1%), whereas healthy normal bone tissues displayed minimal expression (12.5%, *p* < 0.001). These findings align with those of Murray et al. [12], who used immunohistochemistry to demonstrate CYP1B1 overexpression in 67% of the osteosarcoma samples. Such slight variations in CYP1B1 expression rates may be attributed to differences in the cohorts used, detection thresholds, and methodological protocols. Other malignancies, such as lung, prostate, bladder, ovarian, colorectal, and breast cancers, have also been shown to overexpress CYP1B1. Its high expression is frequently associated with tumor development, drug resistance, and unfavorable prognosis, making it a potential therapeutic target and biomarker [1,2,13].

In support of these conclusions, an independent analysis of RNA-seq data revealed that osteosarcoma tissues had much lower levels of CYP1B1 mRNA than normal bone tissues. Such discrepancies between mRNA and protein expression suggest involvement of post-transcriptional and translational regulatory mechanisms. This involvement has been supported by several studies. The expression of CYP1B1 can be epigenetically regulated in cancer cells. Therapy with demethylating drugs such as 5-aza-2′-deoxycytidine restores its expression, while promoter methylation inhibits its transcription [14]. Moreover, MicroRNAs, like miR-27b and miR-187, specifically target CYP1B1 mRNA, decreasing the stability of transcription and efficiency of translation. The dysregulation of these microRNAs in malignancies may consequently lead to the accumulation of CYP1B1 protein despite decreased transcript levels [15,16,17,18]. Additionally, higher protein levels may also be explained by decreased CYP1B1 degradation or improved protein stability in cancerous cells [4]. Collectively, the difference between transcript and protein levels highlights the significance of assessing both mRNA and protein expression when analyzing biomarkers. Consistent CYP1B1 protein detection across studies supports its diagnostic and therapeutic potential.

Based on the expression data, we investigated the association between CYP1B1protein expression and clinical characteristics, such as demographic and prognostic variables. CYP1B1 expression was significantly correlated with age in patients below 34 years (*p* = 0.013), which aligns with the prevalence of osteosarcoma in adolescents and young adults [8]. This is particularly true because CYP1B1 is an estrogen-regulated protein, and hormone-driven processes during skeletal development may be the cause of its elevated expression in younger people [19,20]. Although the survival of CYP1B1-positive patients was slightly lower (mean 58.8 months) than that of CYP1B1-negative patients (62.8 months), this difference was not statistically significant (*p* = 0.170). Interestingly, univariate analysis revealed a significant correlation between CYP1B1 expression and worse prognosis (HR = 1.335, *p* = 0.039). However, this correlation was not retained in the multivariable model (HR = 1.188, *p* = 0.229), as tumor grade (HR = 2.227), lymph node involvement (HR = 33.04), and distant metastasis (HR = 32.19) were more dominant predictors. These findings suggest that CYP1B1 expression may mirror tumor aggressiveness but does not function as an independent prognostic biomarker in bone sarcomas. Similar results have been observed for various types of cancer. CYP1B1 is aberrantly overexpressed and associated with carcinogenesis, drug metabolism, and progression. Nevertheless, its association with patient survival or prognosis is uncertain. For instance, it was linked to adverse features in advanced lung cancers, but there was no clear association with overall survival [21]. Similarly, CYP1B1 was found to promote tumorigenicity in renal cell carcinoma, but no significant correlation was found with overall survival [22,23]. Therefore, CYP1B1 seems to be more predictive of tumor development than an independent predictor of prognosis.

By analogy to other cancers, CYP1B1 may promote bone sarcoma development via several oncogenic mechanisms. By downregulating E-cadherin and upregulating transcription factors such as ZEB2, SNAI1, and TWIST1, CYP1B1 activates epithelial–mesenchymal transition (EMT), promoting cellular migration and invasion [24,25]. Additionally, via Sp1 induction, CYP1B1 regulates Wnt/β-catenin signaling, resulting in β-catenin nuclear accumulation and, subsequently, the overexpression of oncogenes, such as cyclin D1 and c-Myc [24,26]. Moreover, CYP1B1 may induce DNA damage and trigger neoplastic transformation in the bone microenvironment by converting estradiol into carcinogenic 4-hydroxyestradiol (4-OHE2) through the estrogen metabolism pathway [24,27]. Furthermore, CYP1B1 hinders apoptosis by inhibiting the activation of caspase-1 (CASP1) and downregulating pro-apoptotic DAPK1, thereby providing tumor cells with a survival advantage [26,27].

Given the observed expression trends, it is critical to explore the possible regulatory mechanisms that may trigger CYP1B1 overexpression in bone sarcomas. Aryl hydrocarbon receptor (AhR) ligands, such as TCDD, activate xenobiotic response elements (XREs) in the promoter of CYP1B1, making the AhR pathway a crucial regulator of CYP1B1 expression [28,29]. This has been observed in osteosarcoma cell lines, where elevated CYP1B1 mRNA and protein levels result from AhR pathway activation. Hormonal regulation also plays a role; in osteosarcoma cell models, treatment with 17β-estradiol leads to an increase in the expression of CYP1B1 [2,30]. Furthermore, CYP1B1 mRNA stability and translation are post-transcriptionally modulated by microRNAs such as miR-27b and miR-187 [16,31]. These data indicate the complex and dynamic regulation of CYP1B1 in bone sarcomas, which is consistent with the PPI map’s networked functions showing CYP1B1 as a key hub connecting detoxification, hormone, and xenobiotic metabolism through the cooperation of UGT family proteins, COMT, GSTP1, and others. From a clinical perspective, this analysis should be considered as exploratory and hypothesis-generating rather than giving direct therapeutic implications. Its significance resides in recognizing potential cascades and molecular interactions that require mechanistic validation in experimental sarcoma models. Consequently, the PPI data encourage further investigations but should not be regarded as indicative of direct therapeutic significance.

These CYP1B1 expression patterns and mechanistic insights may have substantial diagnostic and therapeutic implications for bone sarcomas. Despite CYP1B1’s poor prognostic value, its consistent selective expression makes it a potential candidate for the development of targeted therapies for bone sarcomas. Targeted therapies leveraging CYP1B1 activity, such as prodrug systems or immune-based approaches, may improve therapeutic specificity with less systemic toxicity [30,31]. Additionally, the potential of RNA interference techniques and small-molecule compounds targeting CYP1B1 activity is being assessed to improve chemosensitivity and overcome chemotherapy resistance [32,33]. This aligns with our drug sensitivity analysis, which demonstrated mild positive (resistance) and negative (response) correlations between CYP1B1 expression and various drugs across pan-cancer GDSC datasets. Although interesting, the correlation between CYP1B1 expression and drug sensitivity in the GDSC dataset should be interpreted with caution. These results are exploratory and hypothesis-generating, suggesting that CYP1B1 modulation may have therapeutic implications, particularly with EGFR/AKT-targeting drugs and HDAC inhibitors. Collectively, these findings support the integration of CYP1B1-targeted strategies into therapeutic pipelines, pending validation in future preclinical and clinical studies, particularly for bone sarcomas.

The study has several limitations. RNA and protein data were collected from unmatched cohorts, rendering the explanation of post-transcriptional regulation speculative. Only a small osteosarcoma cohort without any chondrosarcoma cases had transcriptomic data, and CYP1B1 protein expression was evaluated only by immunohistochemistry without orthogonal validation. The survival analysis comprised only 78 patients, including a small chondrosarcoma subgroup, which reduced statistical power. Finally, drug sensitivity data were exploratory pan-cancer correlations and, without further validation in sarcoma cell lines, should not be regarded as predictive for bone sarcomas.

## 4. Materials and Methods

### 4.1. Tissue Specimens and Immunohistochemistry

Before the start of this investigation, ethical approval was obtained from the Institutional Review and Ethics Committee, Faculty of Medicine, Mutah University (Reference No. 43025, date: 15 June 2025). A customized immunohistochemistry protocol was developed for the analysis of wax-embedded tissue microarray (TMA) sections, which integrated several modifications to improve specificity and maintain structural integrity. The TMAs were for osteosarcoma and chondrosarcoma tissue arrays with survival data, pathology grade, TNM classification, and clinical stage (OSCHS801MSur; TissueArray.com LLC, Derwood, MD, USA). To enable comparative analysis, cancer-adjacent normal bone and cartilage tissue arrays were included in the study (BO2081 and T261b; TissueArray.Com LLC). The inclusion criteria were histologically validated osteosarcoma and chondrosarcoma samples, together with available clinicopathological data (tumor size, grade, stage, and metastasis) and survival information. Normal bone and cartilage tissues from non-malignant donors were utilized as controls. Moreover, tissues with damaged or low-quality cores, inadequate clinical annotation, or no histological confirmation were excluded. At the start, each TMA section was treated with an organic solvent by immersing it in a xylene-based clearing solution. This was followed by a rehydration gradient that utilized progressively decreasing concentrations of ethanol, ending with distilled water. To expose masked antigenic sites, the heat-induced epitope retrieval (HIER) method was used by microwaving (650 W) the TMAs in citrate buffer (pH 6.0) for 20 min. The slides were then allowed to cool to room temperature and rinsed with phosphate-buffered saline (PBS). Subsequently, the activity of endogenous peroxidase was inhibited by applying 3% hydrogen peroxide for 5 min. To minimize nonspecific binding, the slides were incubated with 5% normal serum for 30 min at room temperature. A CYP1B1 rabbit polyclonal antibody (NBP1-85496, Novus Biological, Centennial, CO, USA) was applied at an optimized concentration of 10 μg/mL and incubated for 1 h at room temperature. Following a series of PBS washes, the tissue arrays were incubated with a specific polymer goat anti-rabbit IgG reagent (ImmPRESS, MP-7451; Vector Laboratories, Newark, CA, USA) for 30 min. Immunoreactivity was detected using 3,3′-diaminobenzidine (DAB) substrate for 3–5 min. To achieve sharp, high-contrast signals, the tissue arrays were selectively counterstained with diluted Mayer hematoxylin. Finally, the tissue arrays were dehydrated, air-dried, and mounted using low-viscosity permanent mounting resin. To ensure the specificity of staining, negative controls were performed by omitting the CYP1B1 primary antibody and neutralizing the CYP1B1 primary antibody through pre-incubation with the corresponding peptide (NBP1-85496PEP). Colon cancer tissue was used as a positive control for staining.

### 4.2. Scoring of CYP1B1 Expression

The immunohistochemical staining results were assessed using the Allred scoring system, which offers a semi-quantitative evaluation by considering two criteria: the percentage of positively stained cancer cells and staining intensity [34,35]. Each percentage score was designated as follows: 0 for the absence of positive cells, 1 for less than 1%, 2 for 1–10%, 3 for 11–33%, 4 for 34–66%, and 5 for 67–100% of the positively stained cells. The intensity score was quantified according to the mean staining intensity in cancer cells, rated as 0 (no staining), 1 (weak staining), 2 (moderate staining), and 3 (strong staining). The absolute Allred score was determined by summing the percentage and intensity scores, resulting in a total score of 0–8. For marker expression, scores ranging from 0 to 2 were considered negative, and those ranging from 3 to 8 were considered positive. This scoring method offers a standardized approach for evaluating protein expression and is commonly used to analyze biomarkers in solid malignancies [36,37,38]. IHC scoring was independently performed by two experienced pathologists, and discrepancies were resolved by consensus.

### 4.3. CYP1B1 Gene Expression

Transcriptomic profiles for osteosarcoma and normal samples were retrieved from the Gene Expression Omnibus (GEO) under accession GSE126209 using the website interface portal and the GEOquery Bioconductor package [39]. Unfortunately, publicly available transcriptomic datasets for chondrosarcoma were not accessible, limiting RNA expression analysis to osteosarcoma. The dataset comprised 22 samples (10 normal and 12 osteosarcoma samples). Raw counts for CYP1B1 were extracted and log2-transformed as log2(count + 1) to stabilize variance and reduce skewness. For each condition, we computed the mean log2-expression and plotted these values using the ggplot2 package. Welch’s *t*-test was employed to assess the statistical significance of the differences in gene expression between osteosarcoma and normal samples. This test is a robust alternative to the standard Student’s *t*-test, does not assume equal variances between groups, and handles type 1 error better than Student’s *t*-test, making it particularly suitable for comparisons involving unequal sample sizes or heterogeneous variances, as in this study.

### 4.4. Protein–Protein Interactions (PPI) Network

To elucidate the interaction profile of CYP1B1, we constructed a protein–protein interaction (PPI) network using STRING version 12 (https://string-db.org/). The analysis was restricted to Homo sapiens, with a minimum required interaction score of 0.7 (high confidence). These interactions include both physical binding and functional association. K-means clustering was performed to identify functional modules within the network, with the number of clusters set to three.

### 4.5. Structural Prediction of Canonical CYP1B1 Using AlphaFold

The three-dimensional structure of CYP1B1 was retrieved from the AlphaFold Protein Structure Database. This model corresponds to the canonical isoform of human CYP1B1, as confirmed by UniProt accession number Q16678. The structure was generated using the AlphaFold Monomer v2.0 pipeline (AlphaFold DB version 2022-11-01), and the full-length protein contained 543 amino acids. Per-residue confidence scores (pLDDT) were used to assess structural accuracy, with residues scoring above 90 being considered as highly reliable [40,41].

### 4.6. Drug Sensitivity Correlation with CYP1B1 Expression

To investigate the association between CYP1B1 mRNA expression and drug response in pan-cancer, we used the Genomics of Drug Sensitivity in Cancer (GDSC) dataset. Gene expression profiles and drug sensitivity data (IC50 values) were obtained from the GDSC. The results were generated using Gene Set Cancer Analysis [42].

### 4.7. Statistical Analysis

SPSS 25 (IBM, Armonk, NY, USA, version 20) was used to analyze the data, and frequency and percentage values were used to present the results. The Chi-square test, Fisher’s exact test, and one-way analysis of variance (ANOVA) were used, when applicable, to determine the differences between the measured variables. The Kaplan–Meier method was used to assess patient survival, and the log-rank test was used to calculate the statistical significance between survival means. Both univariate and multivariate Cox regression analyses were performed to determine the prognostic potential of CYP1B1. Differences were considered statistically significant at *p* < 0.05.

## 5. Conclusions

This study revealed a consistent and selective overexpression of CYP1B1 protein in bone sarcomas, particularly osteosarcoma and chondrosarcoma, compared to normal bone tissue. This overexpression is more prominent in younger patients and may reflect hormonally driven or epigenetically regulated mechanisms of tumorigenesis. Its overexpression in bone sarcomas may mirror tumor aggressiveness, but not an independent prognostic biomarker. Its main potential lies in serving as a diagnostic marker or therapeutic target rather than as a survival predictor. The discrepancy between low mRNA and high protein levels suggests complex post-transcriptional regulation, potentially involving microRNAs and RNA stability factors. The protein–protein interaction network further underscores CYP1B1’s centrality in xenobiotic metabolism, estrogen regulation, and oxidative stress pathways, which are hallmarks of tumor biology.

Importantly, CYP1B1 exhibited differential correlations with drug sensitivity, including both resistance and responsiveness to specific agents, such as HDAC inhibitors and EGFR-targeting compounds. This indicates its value as a biomarker and a target for personalized therapeutic strategies. CYP1B1-targeted modalities, including prodrug systems, RNA interference, small-molecule inhibitors, and immunotherapies, may improve treatment specificity and efficacy while minimizing toxicity. Given its selective expression, regulatory complexity, and interaction with key oncogenic pathways, CYP1B1 is a promising target for therapeutic exploration. Future in vitro and in vivo validation and clinical trials are warranted to establish its utility as a therapeutic target or companion diagnostic tool, particularly in aggressive, relapsed, or chemoresistant bone sarcomas.

## Figures and Tables

**Table 1:** Illustration of sociodemographic and clinicopathologic features of both CYP1B1-negative and positive groups.

	CYP1B1		*p*-Value
	Negative (*n* = 26, 27.7%)	Positive (*n* = 68, 72.3%)	
**Age**			
≤34 (*n* = 52, 55.3%)	11 (21.2%)	41 (78.8%)	0.013 ^Χ^
>34 (*n* = 42, 44.7%)	19 (45.2%)	23 (54.8%)	
**Sex**			
Male (*n* = 62, 66.0%)	17 (27.4%)	45 (72.6%)	0.193 ^Χ^
Female (*n* = 32, 34.0%)	13 (40.6%)	19 (59.4%)	
**Diagnosis**			
Osteosarcoma (*n* = 50, 53.2%)	11 (22.0%)	39 (78.0%)	0.001 ^Χ^
Chondrosarcoma (*n* = 28, 29.8%)	5 (17.9%)	23 (82.1%)	
Normal tissue (*n* = 16, 17.0%)	14 (87.5%)	2 (12.5%)	
**Tumor size**			
T1 (*n* = 20, 25.6%)	5 (25.0%)	15 (75.0%)	0.540 *
T2 (*n* = 58, 74.4%)	11 (19.0%)	47 (81.0%)	
**Lymph node metastasis**			
Negative (*n* = 76, 97.4%)	16 (21.1%)	60 (78.9%)	1.000 *
Positive (*n* = 2, 2.6%)	0 (0.0%)	2 (100.0%)	
**Distant metastasis**			
Negative (*n* = 75, 96.2%)	16 (21.3%)	59 (78.7%)	1.000 *
Positive (*n* = 3, 3.8%)	0 (0.0%)	3 (100.0%)	
**Histological grade**			
Grade 1 (*n* = 23, 29.5%)	6 (26.1%)	17 (73.9%)	0.540 *
Grade 3 (*n* = 55, 70.5%)	10 (18.2%)	45 (81.8%)	
**Clinical Stage**			
Stage 1 (*n* = 23, 29.5%)	6 (26.1%)	17 (73.9%)	0.420 ^Χ^
Stage 2 (*n* = 50, 64.1%)	10 (20.0%)	40 (80.0%)	
Stage 4 (*n* = 5, 6.4%)	0 (0.0%)	5 (100.0%)	

^Χ^: Chi-square test of independence; *: Fisher’s exact test.

**Table 2:** Univariable and Multivariable Cox regression models highlighting the prognostic value of several clinicopathologic features, and the CYP1B1 expression status.

	Univariable			Multivariable		
	HR	95% CI	*p*-Value	HR	95% CI	*p*-Value
**Age**	1.025	1.003–1.047	0.027	1.040	1.017–1.064	0.001
**Sex**	0.775	0.383–1.570	0.480	0.694	0.333–1.445	0.329
**Diagnosis**	0.798	0.393–1.620	0.533	1.369	0.531–3.530	0.516
**T**	2.132	0.830–5.480	0.116	2.709	1.005–7.301	0.049
**N**	33.042	5.971–182.837	<0.001	N/A		
**M**	32.187	6.453–160.544	<0.001	N/A		
**Grade**	1.695	1.058–2.716	0.028	2.227	1.227–4.041	0.008
**Stage**	5.255	2.671–10.339	<0.001	N/A		
**CYP1B1**	1.335	1.014–1.758	0.039	1.188	0.897–1.573	0.229

(HR = Hazard Ratio; CI = Confidence Interval; T = Tumor size; N = Nodal status; M = Metastasis).

## Data Availability

The data presented in this study are available on request from the corresponding author. The data are not publicly available due to privacy and ethical concerns.

## Abbreviations

The following abbreviations are used in this manuscript:

CYP1B1	Cytochrome 1B1
TMA	tissue microarray
HIER	heat-induced epitope retrieval
PBS	phosphate-buffered saline
DAB	3,3′-diaminobenzidine
GEO	Gene Expression Omnibus
ANOVA	one-way analysis of variance
EMT	epithelial–mesenchymal transition
AhR	aryl hydrocarbon receptor
XREs	xenobiotic response elements

## References

[1] Shimada T., Fujii-Kuriyama Y. (2004). Metabolic activation of polycyclic aromatic hydrocarbons to carcinogens by cytochromes P450 1A1 and 1B1. Cancer Sci..

[2] Hayes C.L., Spink D.C., Spink B.C., Cao J.Q., Walker N.J., Sutter T.R. (1996). 17 beta-estradiol hydroxylation catalyzed by human cytochrome P450 1B1. Proc. Natl. Acad. Sci. USA.

[3] D’Uva G., Baci D., Albini A., Noonan D.M. (2018). Cancer chemoprevention revisited: Cytochrome P450 family 1B1 as a target in the tumor and the microenvironment. Cancer Treat. Rev..

[4] Murray G.I., Melvin W.T., Greenlee W.F., Burke M.D. (2001). Regulation, function, and tissue-specific expression of cytochrome P450 CYP1B1. Annu. Rev. Pharmacol. Toxicol..

[5] Alshammari F.O.F.O., Al-Saraireh Y.M., Youssef A.M.M., Al-Sarayra Y.M., Alrawashdeh H.M. (2021). Cytochrome P450 1B1 Overexpression in Cervical Cancers: Cross-sectional Study. Interact. J. Med. Res..

[6] Al-Saraireh Y.M., Alshammari F., Youssef A.M.M., Al-Sarayreh S., Almuhaisen G.H., Alnawaiseh N., Al Shuneigat J.M., Alrawashdeh H.M. (2021). Profiling of CYP4Z1 and CYP1B1 expression in bladder cancers. Sci. Rep..

[7] Society A.C. Key Statistics for Bone Cancer. https://www.cancer.org/cancer/types/bone-cancer/about/key-statistics.html.

[8] Strauss S.J., Frezza A.M., Abecassis N., Bajpai J., Bauer S., Biagini R., Bielack S., Blay J.Y., Bolle S., Bonvalot S. (2021). Bone sarcomas: ESMO–EURACAN–GENTURIS–ERN PaedCan Clinical Practice Guideline for diagnosis, treatment and follow-up. Ann. Oncol..

[9] Marina N., Gebhardt M., Teot L., Gorlick R. (2004). Biology and therapeutic advances for pediatric osteosarcoma. Oncologist.

[10] Franchi A. (2012). Epidemiology and classification of bone tumors. Clin. Cases Miner. Bone Metab..

[11] Italiano A., Mir O., Cioffi A., Palmerini E., Piperno-Neumann S., Perrin C., Chaigneau L., Penel N., Duffaud F., Kurtz J.E. (2013). Advanced chondrosarcomas: Role of chemotherapy and survival. Ann. Oncol..

[12] Dhaini H.R., Thomas D.G., Giordano T.J., Johnson T.D., Biermann J.S., Leu K., Hollenberg P.F., Baker L.H. (2003). Cytochrome P450 CYP3A4/5 expression as a biomarker of outcome in osteosarcoma. J. Clin. Oncol..

[13] Falero-Perez J., Song Y.S., Sorenson C.M., Sheibani N. (2018). CYP1B1: A key regulator of redox homeostasis. Trends Cell Mol. Biol..

[14] Habano W., Gamo T., Sugai T., Otsuka K., Wakabayashi G., Ozawa S. (2009). CYP1B1, but not CYP1A1, is downregulated by promoter methylation in colorectal cancers. Int. J. Oncol..

[15] Tsuchiya Y., Nakajima M., Takagi S., Taniya T., Yokoi T. (2006). MicroRNA regulates the expression of human cytochrome P450 1B1. Cancer Res..

[16] Szczepanek J., Skorupa M., Tretyn A. (2022). MicroRNA as a Potential Therapeutic Molecule in Cancer. Cells.

[17] Buddingh E.P., Kuijjer M.L., Duim R.A., Bürger H., Agelopoulos K., Myklebost O., Serra M., Mertens F., Hogendoorn P.C., Lankester A.C. (2011). Tumor-infiltrating macrophages are associated with metastasis suppression in high-grade osteosarcoma: A rationale for treatment with macrophage activating agents. Clin. Cancer Res..

[18] Kuijjer M.L., Rydbeck H., Kresse S.H., Buddingh E.P., Lid A.B., Roelofs H., Bürger H., Myklebost O., Hogendoorn P.C., Meza-Zepeda L.A. (2012). Identification of osteosarcoma driver genes by integrative analysis of copy number and gene expression data. Genes. Chromosomes Cancer.

[19] Li F., Zhu W., Gonzalez F.J. (2017). Potential role of CYP1B1 in the development and treatment of metabolic diseases. Pharmacol. Ther..

[20] Shah B.R., Xu W., Mraz J. (2019). Cytochrome P450 1B1: Role in health and disease and effect of nutrition on its expression. RSC Adv..

[21] Su J.-M., Lin P., Wang C.-K., Chang H. (2009). Overexpression of Cytochrome P450 1B1 in Advanced Non-small Cell Lung Cancer: A Potential Therapeutic Target. Anticancer Res..

[22] Chang I., Mitsui Y., Fukuhara S., Gill A., Wong D.K., Yamamura S., Shahryari V., Tabatabai Z.L., Dahiya R., Shin D.M. (2015). Loss of miR-200c up-regulates CYP1B1 and confers docetaxel resistance in renal cell carcinoma. Oncotarget.

[23] Kwon Y.J., Baek H.S., Ye D.J., Shin S., Kim D., Chun Y.J. (2016). CYP1B1 Enhances Cell Proliferation and Metastasis through Induction of EMT and Activation of Wnt/β-Catenin Signaling via Sp1 Upregulation. PLoS ONE.

[24] Zhang J., Wang W. (2025). Glypican-3 regulated epithelial mesenchymal transformation-related genes in osteosarcoma: Based on comprehensive tumor microenvironment profiling. Front. Immunol..

[25] Mitsui Y., Chang I., Fukuhara S., Hiraki M., Arichi N., Yasumoto H., Hirata H., Yamamura S., Shahryari V., Deng G. (2015). CYP1B1 promotes tumorigenesis via altered expression of CDC20 and DAPK1 genes in renal cell carcinoma. BMC Cancer.

[26] Chang I., Mitsui Y., Kim S.K., Sun J.S., Jeon H.S., Kang J.Y., Kang N.J., Fukuhara S., Gill A., Shahryari V. (2017). Cytochrome P450 1B1 inhibition suppresses tumorigenicity of prostate cancer via caspase-1 activation. Oncotarget.

[27] Denison M.S., Soshilov A.A., He G., DeGroot D.E., Zhao B. (2011). Exactly the same but different: Promiscuity and diversity in the molecular mechanisms of action of the aryl hydrocarbon (dioxin) receptor. Toxicol. Sci..

[28] Murray I.A., Patterson A.D., Perdew G.H. (2014). Aryl hydrocarbon receptor ligands in cancer: Friend and foe. Nat. Rev. Cancer.

[29] Deb S., Tai J.K., Leung G.S., Chang T.K., Bandiera S.M. (2011). Estradiol-mediated suppression of CYP1B1 expression in mouse MA-10 Leydig cells is independent of protein kinase A and estrogen receptor. Mol. Cell. Biochem..

[30] McFadyen M.C., Melvin W.T., Murray G.I. (2004). Cytochrome P450 enzymes: Novel options for cancer therapeutics. Mol. Cancer Ther..

[31] Yuan B., Liu G., Dai Z., Wang L., Lin B., Zhang J. (2022). CYP1B1: A Novel Molecular Biomarker Predicts Molecular Subtype, Tumor Microenvironment, and Immune Response in 33 Cancers. Cancers.

[32] Martinez V.G., O’Connor R., Liang Y., Clynes M. (2008). CYP1B1 expression is induced by docetaxel: Effect on cell viability and drug resistance. Br. J. Cancer.

[33] Mikstacka R., Dutkiewicz Z. (2021). New Perspectives of CYP1B1 Inhibitors in the Light of Molecular Studies. Processes.

[34] Harvey J.M., Clark G.M., Osborne C.K., Allred D.C. (1999). Estrogen receptor status by immunohistochemistry is superior to the ligand-binding assay for predicting response to adjuvant endocrine therapy in breast cancer. J. Clin. Oncol..

[35] Al-Saraireh Y.M., Alshammari F., Youssef A.M.M., Al-Sarayreh S., Almuhaisen G.H., Alnawaiseh N., Al-Shuneigat J.M., Alrawashdeh H.M. (2021). Cytochrome 4Z1 Expression is Associated with Poor Prognosis in Colon Cancer Patients. OncoTargets Ther..

[36] Alshammari F., Al-Saraireh Y.M., Youssef A.M.M., Al-Sarayra Y.M., Alrawashdeh H.M. (2021). Glypican-1 Overexpression in Different Types of Breast Cancers. OncoTargets Ther..

[37] Al-Saraireh Y.M., Alboaisa N.S., Alrawashdeh H.M., Hamdan O., Al-Sarayreh S., Al-Shuneigat J.M., Nofal M.N. (2020). Screening of cytochrome 4Z1 expression in human non-neoplastic, pre-neoplastic and neoplastic tissues. Ecancermedicalscience.

[38] Alshammari F.O., Satari A.O., Aljabali A.S., Al-mahdy Y.S., Alabdallat Y.J., Al-sarayra Y.M., Alkhojah M.A., Alwardat A.r.M., Haddad M., Al-sarayreh S.A. (2022). Glypican-3 Differentiates Intraductal Carcinoma and Paget’s Disease from Other Types of Breast Cancer. Medicina.

[39] Davis S., Meltzer P.S. (2007). GEOquery: A bridge between the Gene Expression Omnibus (GEO) and BioConductor. Bioinformatics.

[40] Jumper J., Evans R., Pritzel A., Green T., Figurnov M., Ronneberger O., Tunyasuvunakool K., Bates R., Žídek A., Potapenko A. (2021). Highly accurate protein structure prediction with AlphaFold. Nature.

[41] Varadi M., Bertoni D., Magana P., Paramval U., Pidruchna I., Radhakrishnan M., Tsenkov M., Nair S., Mirdita M., Yeo J. (2024). AlphaFold Protein Structure Database in 2024: Providing structure coverage for over 214 million protein sequences. Nucleic Acids Res..

[42] Liu C.-J., Hu F.-F., Xia M.-X., Han L., Zhang Q., Guo A.-Y. (2018). GSCALite: A web server for gene set cancer analysis. Bioinformatics.

